# A Systematic Review on the Implications of O-linked Glycan Branching and Truncating Enzymes on Cancer Progression and Metastasis

**DOI:** 10.3390/cells9020446

**Published:** 2020-02-14

**Authors:** Rohitesh Gupta, Frank Leon, Sanchita Rauth, Surinder K. Batra, Moorthy P. Ponnusamy

**Affiliations:** 1 Department of Biochemistry and Molecular Biology, University of Nebraska Medical Center, Omaha, NE 68105, USA; rohitesh.gupta@gmail.com (R.G.); frank.leon@unmc.edu (F.L.); sanchita.rauth@unmc.edu (S.R.); 2 Fred and Pamela Buffett Cancer Center, Eppley Institute for Research in Cancer and Allied Diseases, University of Nebraska Medical Center, Omaha, NE 681980-5900, USA; 3 Department of Pathology and Microbiology, UNMC, Omaha, NE 68198-5900, USA

**Keywords:** glycosylation, cancer, metastasis

## Abstract

Glycosylation is the most commonly occurring post-translational modifications, and is believed to modify over 50% of all proteins. The process of glycan modification is directed by different glycosyltransferases, depending on the cell in which it is expressed. These small carbohydrate molecules consist of multiple glycan families that facilitate cell–cell interactions, protein interactions, and downstream signaling. An alteration of several types of O-glycan core structures have been implicated in multiple cancers, largely due to differential glycosyltransferase expression or activity. Consequently, aberrant O-linked glycosylation has been extensively demonstrated to affect biological function and protein integrity that directly result in cancer growth and progression of several diseases. Herein, we provide a comprehensive review of several initiating enzymes involved in the synthesis of O-linked glycosylation that significantly contribute to a number of different cancers.

## 1. Introduction

Glycosylation is the most complex and ubiquitous post-translational modification process found on a variety of secretory and membrane-bound proteins [1]. Glycosylation occurs because of catalytic action by enzymes termed glycosyltransferases. These enzymes are mostly specific to the endoplasmic reticulum (ER), Golgi, and the extracellular milieu [2,3,4]. Reactions that are catalyzed by glycosyltransferases occur through a bi-bi substrate mechanism, wherein a sugar-nucleotide donor and a carbohydrate acceptor participate to form a modified glycan and a nucleoside as the products [5]. The complexity in glycosylation occurs due to the inherent stochasticity arising from the enzyme concentration, transport phenomenon, kinetic parameters, thermodynamics, and sugar-nucleotide transporters [6,7]. These and many other parameters altogether govern the associated micro- and macro-heterogeneity in glycosylation processes [8,9,10]. In humans, various types of glycosylation are found, including, but not limited to, O-linked and N-linked glycosylation [11,12,13]. N-glycosylation occurs from the transfer of N-acetyl glucosamine phosphate to the dolichol phosphate. A variety of glycosyltransferases that are localized in the ER result in an extension of N-glycosylation. The N-linked carbohydrates then mature further by the action of Golgi-resident glycosyltransferase [14]. For N-glycosylation, oligosaccharides are attached to asparagine (N) of a tripeptide consensus sequence of N-X-S/T. Therefore, the polypeptide on which it can be found is known, and the study of N-glycosylation is easier compared to O-glycosylation [15]. However, Golgi-resident enzymes initiate and extend O-glycosylation, as opposed to the N-glycosylation, which initiates in the ER. Both O- and N-glycosylation participate in cancer biology and have gained much attention in recent years [16]. In this review, we will primarily discuss the role of initiating O-linked glycosylation and its effects on cancer progression and metastasis [17]. 

## 2. O-Glycosylation

O-linked glycosylation is considered more complicated than N-linked as the consensus sequence for its initiation is unknown. Moreover, most of the mucinous proteins are decorated by hundreds of O-linked glycosylation patterns, making its role important in many cancer-associated biological processes [18]. The initiation of O-glycosylation occurs via the addition of N-acetylgalactosamine (GalNAc) on the serine/threonine (Ser/Thr) residue of the protein backbone. This addition is catalyzed by N-acetylgalactosaminyltransferase (GALNT) enzymes, of which 20 isoforms are known in humans [19]. Following the addition of GalNAc, either galactose (Gal) or N-acetylglucosamine (GlcNAc) addition occurs onto the resultant monosaccharide via the action of C1GALT1 (Core 1 Synthase, Glycoprotein-N-Acetylgalactosamine 3-Beta-Galactosyltransferase 1; herein referred to as Core-1 synthase) or B3GNT6 (UDP-GlcNAc:BetaGal Beta-1,3-N-Acetylglucosaminyltransferase 6; herein referred to as Core-3 synthase), respectively [20]. In conditions where the expression of ST6GalNAc (ST6 N-Acetylgalactosaminide Alpha-2,6-Sialyltransferase 1) enzyme is high, N-acetylneuraminic acid (sialic acid) acts on the GalNAc moiety, resulting in the truncation of O-glycan by forming Neu5Acα2,6GalNAc (Sialyl-Tn) [21]. The formation of sialyl-Tn results in O-glycan abrogation and has implications in cancer [22]. ST6GalNAc-I is an enzyme belonging to the class of sialyltransferases and possesses four motifs on its polypeptide: namely, L (long), S (short), III, and VS (Very Small). These motifs bind to either the donor or an acceptor. Currently, no crystal structures are available for these sialyltransferases and thus their binding specificity is unknown. However, the function of ST6GalNAc-I results in the truncation of O-glycans by competing with Core-2 GlcNAc transferase [23]. In human systems, there are three Core 2 GlcNAc transferases, C2GnT-1 (GCNT1), C2GnT-2 (GCNT2), and C2GnT-3 (GCNT3) [24]. Among the Core-2 glycosyltransferases, GCNT1 and GCNT3 independently attach GlcNAc to the Core-1 and/or Core-3 glycan, resulting in the branching and extension of O-glycosylation [25,26,27]. Core-2 synthase also competes with ST3Gal-I and further truncates O-glycosylation by capping T-antigen with sialic acid. Uncapped T-antigen makes a substrate for Core-2 synthase and thus competition by ST3Gal-I disrupts its function [28]. Moreover, Golgi localization between ST3Gal-I and C2GnT-I has been shown to influence O-glycosylation [29]. 

**Figure 1:**
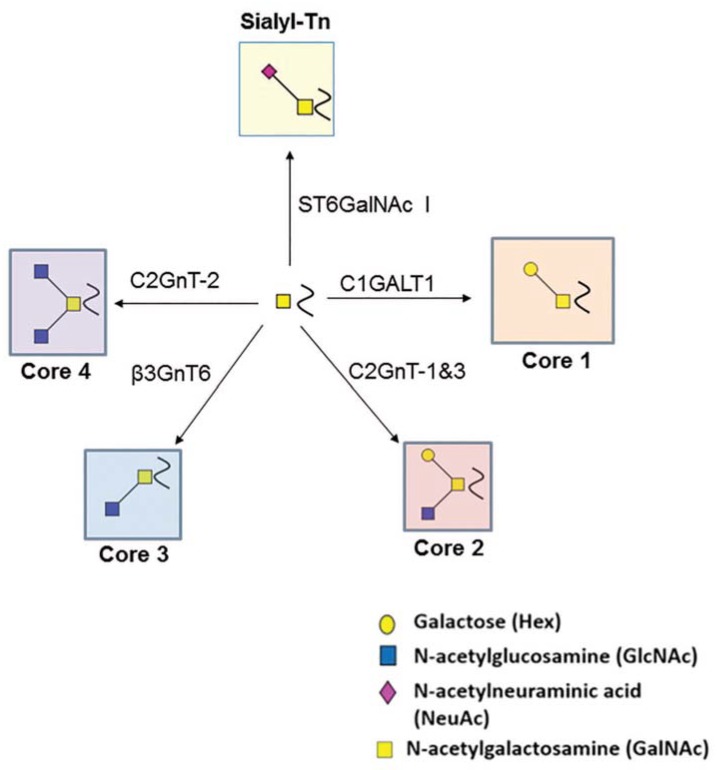
This schematic represents an extension of the O-linked glycans by different initiating and truncating enzymes. Herein, C1GALT1 which is a Core-1 synthase results in the formation of Galβ1,3GalNAc glycan by transferring Galactose from UDP-Gal to GalNAc. Once this enzyme adds galactose, then the substrate becomes favorable for Core-2 formation via C2GnT. This enzyme forms GlcNAcβ1,6(Galβ1,3)GalNAc by transferring GlcNAc (N-acetylglucosamine) from UDP-GlcNAc onto Galβ1,3GalNAc. C1GALT1 competes with ST6GalNAc-I for the addition of Neu5Ac (sialic acid) from CMP-Neu5Ac onto GalNAc. Lastly, C1GALT1 competes with B3GNT6 to form Core-3 structure by adding GlcNAc in a β1,3 linkage onto GalNAc. This enzyme is followed by C2GnT that adds GlcNAc in a β1,6 linkage onto GlcNAcβ1,3GalNAc forming trisaccharide Core-4 structure.

The relative expression of C1GALT1, ST6GalNAc-I, and B3GNT6 enzymes in human systems and their K_M_ (Michaelis-Menten Constant) towards the O-GalNAc substrate determines the variable synthesis of Core-1, -2, -3, and -4 O-glycans. Similarly, the synthesis of Core-1 and Core-3 glycans act as required precursors for the formation of Core-2 and Core-4 glycan synthesis, respectively [30]. The glycosyltransferases mediating O-linked protein modification are important in many biological processes and are largely implicated in several cancers. Overall, O-glycan extension is regulated by C1GalT1, C2GnT, Core-3 synthase, and truncation occurs mainly due to the catalytic action of ST6GalNAc-I. We provide a graphical summary of O-linked modification and the glycosyltransferases that are required for their formation (Figure 1).

## 3. Implications of Key O-Glycosyltransferases

The branching of O-glycosyltransferases, such as C1GALT1, GCNTs, and B3GNT6, results in the formation of Core branches on glycoproteins, thereby modifying protein function and changing biological processes. In this review, we have focused on these key enzymes that either extends O-glycan or abrogates it. The extension of O-glycan is known to form structures such as sLex and sLea. sLex has been known to influence cellular rolling and tethering. In chronic inflammation, cells with sLex-type glycan structures bind with platelets/neutrophils and are attracted towards the inflamed endothelium layer. This results in the initial rolling and tethering of cells. Following this rolling, cells bind with endothelium E-selectin and extravasation occurs. Tumor cells undergo the same phenomenon, thereby becoming more and more metastatic. This sLex synthesis makes glycoproteins the ideal binding partner for various ligands to elicit the effects of cancer.

On the other hand, various reports have shown the relevance of sialyl-Tn antigen in cancer biology. ST6GalNAc-I is the primary enzyme that triggers the synthesis of sialyl-Tn antigen, and its expressional variation causes differential synthesis of Sialyl-Tn. The introduction of sialic acid onto GalNAc inhibits the activity of other branching enzymes and abrogates O-glycan. The carbohydrate antigen sialyl-Tn is linked with poor prognosis in cancer patients and its expression is regulated by different mechanisms in different cancers. In this review, we have covered most of the critical articles linked with the role of sialyl-Tn in cancer.

## 4. Biological Function of TF-Antigen 

Earlier studies on T-synthase focused on its in vivo function by deleting this gene in animal models. Xia et al. reported phenotypic changes upon the deletion of T-synthase in a mouse model using a Cre/loxP system. The authors found that embryos, after day 9, had developed hemorrhages in the brain and spinal cord. The T-synthase^−/−^ embryo was high on HPA lectin (a class of carbohydrate recognition proteins specifically for GalNAc) compared to PNA lectin (this protein recognizes TF-antigen), which recognizes Galβ1,3GalNAc-ser/thr. In the absence of T-synthase, Core-3 or sialyl-Tn were not expressed highly in these embryos. Therefore, the global effects of T-synthase knockout (KO) are lethal in animals, which suggests a critical role of C1GALT1 in mammalian development [31]. 

Ju et al. reported mutations in the X chromosome-encoded COSMC gene, a chaperone for T-synthase, in multiple tumor cell lines. COSMC regulates T-synthase activity by binding to it and preventing aggregation and downstream degradation in the ER [32,33]. It has been reported that Tn antigen formation occurs as a result of a loss in T-synthase activity, which corresponds to clinical data found in > 80% of human carcinomas. Modification of Tn/STn antigens are prominent in > 60% of cervical cancers and also correlated with the poor prognosis of the disease. Ju et al. reported a loss of heterozygosity (LOH) or deletion of cosmc as the major cause for the expression of Tn/STn on cervical cancer. Zeng et al. reported the similarities in the promoter region of T-synthase and COSMC, consisting of two GC-rich sequences next to the transcription start site (TSS), although the promoter region lacked a TATA box. Enzymatic activity was reduced when the transcription factor, SP1/3, binding sites were mutated, suggesting regulation by this transcription factor. In addition, methylome analysis predicted hypermethylation of cosmc promoter region in Tn4 cells, where this gene is silenced, which suggests epigenetic regulation of COSMC but not T-synthase [34]. 

## 5. Core-1 Synthase Loss Aggravates Pancreatic Cancer

**Figure 2:**
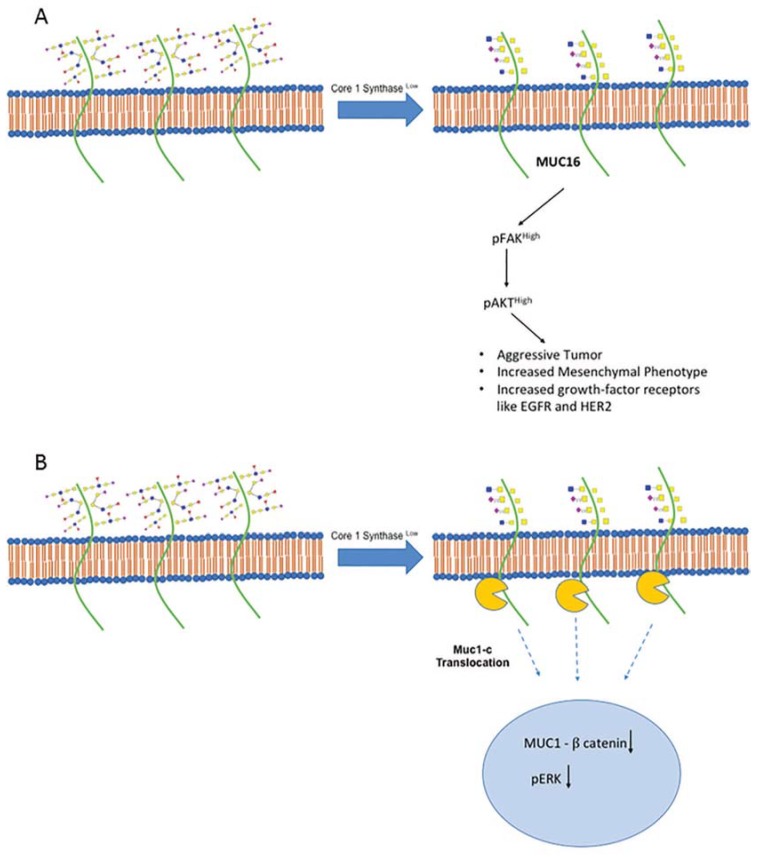
This illustration depicts the findings from a study describing the differential regulation by Core-1 synthase (C1GALT1) on pancreatic cancer (**A**) and breast cancer (**B**). C1GALT1 primarily regulates glycosylation profile of MUC16 in a pancreatic cancer (PC) cell line and in a KPCC mouse model. This aberrant glycosylation of MUC16 then regulates pFAK and pAKT signaling in PC, thereby aggravating tumor and metastasis. This aggressive tumor is also marked by an increase in EMT markers, growth-factor receptors such as EGFR and HER2. On the other hand, C1GALT1 affects MUC-1 glycosylation in breast cancer. This has implications on the transport of MUC1 such that loss of C1GALT1 inhibits MUC1 C-terminus transport to the nucleus that affects downstream β-catenin and pERK signaling.

We recently published a role of C1GALT1 in pancreatic ductal carcinoma using in vitro and in vivo KO models [35]. The deletion of C1GALT1 caused an aggressive phenotype in the context of pancreatic ductal adenocarcinoma. To compare the phenotypic difference of C1GALT1, pancreas-specific deletion of C1GALT1 was introduced, along with Kras and p53 mutation, resulting in a KPCC mouse model (*LSL-Kras^G12D/+^;LSL-Trp53^R172H/+^;Pdx-1-Cre;C1GALT1^−/−^)*. This mouse model resulted in low-grade pancreatic intraepithelial (PanIN) lesions within 3 weeks compared to the KPC (*LSL-Kras^G12D/+^;LSL-Trp53^R172H/+^;Pdx-1-Cre)* model, wherein low-grade PanIN lesions were observed after 5 weeks. The KPCC mouse model had reduced fibrosis as indicated by higher Ki-67 staining in comparison to the KPC mouse model. To assess the severity of C1GALT1 deletion, the metastatic potential to different organs such as the liver, lung, peritoneum, lymph node, diaphragm and stomach was studied. We found that the KPCC tumors metastasized within 10 weeks as compared to 28 weeks for the KPC model. Mechanistically, truncation was observed on the MUC16 O-glycosylation profile with the activation of epithelial-to-mesenchymal transition (EMT) markers. In addition, growth-factor receptors such as EGFR and HER2 were also increased upon the deletion of C1GALT1 in pancreatic cancer cell lines (Figure 2A). Because COSMC influences the function of Core-1 synthase, its role has also been studied in pancreatic cancer progression [36]. This study illustrates that COSMC is regulated through epigenetic silencing and not somatic mutations, resulting in glycan-truncation dependent tumorigenicity. COSMC KO in a T3M4 pancreatic cancer cell line has been shown to induce a proliferative and invasive phenotype. In addition to a pancreatic cancer cell line, a non-tumorigenic keratinocyte specific HaCaT cell line has also been shown to induce a highly tumorigenic phenotype upon deletion of COSMC. Multiomics analysis on HaCaT and T3M4 identified many glycoproteins linked with cellular proliferation and cell–cell adhesion. Overall, studies on T-antigen in the context of pancreatic cancer have suggested an inverse relationship between protein expression and tumor aggression. Both of these studies convincingly suggested that T-antigen synthesis on O-glycan plays an indispensable role in regulating tumor progression and metastasis. Further studies are warranted to delineate the in-depth mechanism of T-antigen’s role in pancreatic cancer. 

## 6. Historical Perspective of Core-1 Synthase in Breast Cancer

Glycosylation changes by Core-1 synthases are evident in tumor progression. Previously, Brockhausen et al. studied the levels of glycosyltransferases in the mammary tumor cell line, MTSV1-7 [37]. The MTSV1-7 cell line decorates glycosylation of MUC1 similar to normal mammary epithelial cells. The group found that while Core-1 synthase activity was comparable in all the cell lines, the C2GnT level was lower in the BT20, MCF-7, and T47D cell lines compared to the MTSV1-7 cell line. Because ST3Gal-I acts downstream of C1GALT1, its levels were also reported, and the authors found eight- to 10-fold higher levels in cancerous cell lines. These glycosylation changes were probed to MUC1 in aforementioned breast cancer cell lines. Because MUC1 is an indispensable mucin involved in breast cancer progression, this study provides direct evidence of the involvement of MUC1 glycosylation in a cancerous tumor conditions. Later, Solatycka et al. also reported an association of MUC1 with T-antigen in breast carcinoma cell lines [38]. The authors indicated overexpression of MUC1 in MDA-MB-231 and T47D cell lines. This resulted in the upregulation of T-antigen and simultaneous downregulation of sLe^x^. The authors also found that there was a decreased enzyme levels of C2GnT1 (GCNT1) and increased levels of ST3Gal-I. However, an overall increase in the expression of T-antigen was associated with MUC1. Thus, the tumor-associated carbohydrate antigen (TACA) present in breast cancer was associated with MUC1. Truncation of Tn and sialyl-Tn antigens are regarded as the TACA for cancer progression. However, many investigators have reported higher expressions of C1GALT1 in breast cancer progression. 

Furthermore, Chou et al. investigated various breast cancer cell lines and studied the role of T-synthase in tumorigenesis [39]. The authors determined that C1GALT1 mRNA and protein levels were found to be higher in breast cancer cell lines and associated with a higher histological grade and tumor stage. In addition, the effect of T-synthase overexpression on in vivo tumor growth in nude mice was studied. Mechanistically, C1GALT1 regulated O-glycans modification on MUC1 and affected MUC1-N shedding and MUC1-C/β-catenin signaling. In C1GALT1 knockdown cells, MUC1-N shedding was markedly decreased, suggesting a role of T-antigen on its extracellular translocation (Figure 2B). In another study highlighting the role of C1GALT1, the authors used an animal model to identify the in vivo role of C1GALT1 in breast cancer [40]. A mammary epithelial specific C1GALT1 KO animal was crossed with MMTV-ErbB2 mice. Upon crossing with the spontaneous tumor-developing animal, there was a reduction in tumor development and lung metastasis. Additionally, C1GALT1 KO mice were crossed with PyMT, resulting in the delayed onset of tumor progression. C1GALT1^−/−^/PyMT mice expressed low Ki67 binding as compared to the control animals, suggesting a decreased proliferation index on mammary epithelial cells. To illustrate the mechanism, MUC-1 was probed in this animal model. The authors found MUC-1 downregulation in C1GALT1^−/−^/PyMT mice, suggesting a possible involvement in the delayed onset of a tumor. MUC-1 downstream signaling molecules, such as Src, PI3k, and Akt, were also downregulated in O-glycan deficient mice, indicating a role in decreased proliferation and tumor suppression. 

While various groups have studied the role of glycosylation in understanding breast cancer biology, the prognostic influence of glycosylation has been seldom studied [41]. The prognostic role of breast cancer reported by Milde-langosch demonstrated that PNA is associated with lymphangiosis, vascular invasion, lymph node involvement, and shorter overall survival (OAS). Because PNA expression is dependent on GALNTs and C1GALT1 activity, the combined prognostic effect of GALNTs and C1GALT1 was reported. It was found that C1GALT1 and GALNT-1, -8, and -14 were associated with an unfavorable prognosis. Thus, this study was the first report to determine the prognostic role of C1GALT1 in breast cancer. 

## 7. Core-1 Synthase Regulates Intestinal Cancer by Different Mechanisms

Core-1- and Core-3-associated O-glycans form a mucus barrier in the gastrointestinal tract, playing an important role in the study of cancer progression. Barrow et al. used two colon cancer cell lines, HT29 and SW620, to knockdown T-synthase and examined competition with ST6GalNAc-I and Core 3-synthase (B3GNT6) [42]. The loss of T-synthase increased antibody binding towards sialyl-Tn and Tn-antigen. Moreover, Griffonia Simplicifolia Lectin II (GSL II), which recognizes α- and β-linked GlcNAc on the non-reducing terminus, also increased, suggesting a higher expression of Core-3 structure. 

To understand the role of colon cancer in physiological conditions, Bergstrom et al. developed animal models in which intestine-specific Core-3 and Core-1 were knocked out in an animal model, termed as double knockout (DKO) [43]. This allowed Bergstrom to study the progression of colon inflammation and tumorigenesis. Their data suggest that a higher expression of Tn antigen is not alone sufficient in inducing tumor formation and may require loss of inflammasome activity to significantly reduce tumor formation, as shown using DKO mice. The authors also discovered that the microbiota was responsible for inflammation and tumorigenesis in DKO mice. Altogether, this study provides a unique model system to address the role of glycosylation in context of the microbiota or the immune system towards different stages of cancer. The same group later studied the role of DKO mice on tumor progression in the small intestine, specifically the duodenum [44]. The spontaneous duodenal tumor occurrence increased in DKO mice as compared to the wild type mice. Upon performing the molecular analysis in DKO mice, impaired luminal expression of Muc-2 was observed. This indicated the role of O-glycans in the formation of the mucus layer. Although the study covers various aspects of O-glycosylation on spontaneous duodenal tumorigenesis, the underlying mechanism is still unknown. Thus, further analysis is required to unravel the role of O-glycans in duodenum tumors. 

Hung et al. explored the role of O-glycosylation in colorectal cancer (CRC) [45]. Specifically, the role of T-synthase was studied in this context and they found that this enzyme was involved in the modification of O-glycan on fibroblast growth factor receptor (FGFR). There are four members of FGFR, namely FGFR-1, -2, -3, and -4. Amongst these, FGFR-2 plays a critical role in the progression of liver, lung, bone and lymph node metastasis, and the aggressiveness of CRC. In this study, the authors found that overexpression of C1GALT1 regulated glycosylation and phosphorylation on FGFR-2, thereby promoting cancer growth. Moreover, there are reports suggesting the downregulation of Core-2 and Core-3 synthase in CRC, thereby implicating the relevance of T-synthase. Dong et al. studied the role of C1GALT1 on CRC and found that its loss increased oncogenic features, such as cell proliferation, invasion, migration, and adhesion [46]. Furthermore, they also found that KO of C1GALT1 resulted in elevated mesenchymal features due to increased protein levels of Snail and Fibronectin and decreased epithelial markers, such as E-cadherin. Because COSMC is known to be the chaperone for C1GALT1, it is an equally important gene to study in the progression of CRC [47]. COSMC expression positively correlated with advanced tumor stages. In addition, the overexpression of COSMC resulted in higher cell proliferation, invasion, and migration amongst the colorectal cell line. The author tested the in vivo role of cancer cells overexpressing COSMC and found higher tumor growth and decreased OAS upon injection in severe combined immunodeficiency (SCID) mice. Knockdown of COSMC decreased malignant behavior in these cell lines by regulating Akt and ERK pathways. In another study, an opposite role of COSMC was observed in CRC [48]. The overexpression of COSMC in LS174T Tn+ cell line caused reduced proliferation, migration, and invasion. In addition, Tn+ cells were found to be more resistant to UV-induced apoptosis as compared to its negative counterpart. This role of T-synthase activity in CRC remains inconsistent with the previous reports and thus demands further assessment. It can be postulated that these results are cell line dependent and do not represent ideal conditions, as one may observe in an animal model or human tissues. 

## 8. Mechanistic Insights of Core-1 Synthase in Hepatocellular Carcinoma

Wu et al. demonstrated an associated expression of T-synthase with an advanced tumor stage, metastasis, and poor prognosis [49]. C1GALT1 also regulated carcinoma cells viability and proliferation in vitro and in vivo. The authors primarily studied the function of MET, which is an oncogene regulating proliferation, poor prognosis, and poor outcome in human hepatocellular carcinoma. The authors confirmed a reduction in cellular viability upon treatment with a MET inhibitor. This activity of MET was shown to be regulated by C1GALT1. In this system, C1GALT1 also induced HGF (Hepatocyte Growth Factor)-dependent dimerization of MET. This study is the first to report the presence of O-glycans on MET and the dependence of its activity on C1GALT1. The same group later considered another perspective of C1GALT1 in hepatocellular carcinoma [50]. Additional glycoproteins other than MET were identified to have a novel mechanism by which T-synthase regulates HCC. The effect of C1GALT1 was exhibited on integrin β1 and its downstream FAK-based signaling events. Because sialyl-Tn and Tn antigens on integrin β1 are known to modulate its activity, the regulatory effects of T-synthase were observed. To this end, the authors found increased activity of integrin β1 due to the expression of T- and sialyl-T antigen on its peptide backbone in C1GALT1 overexpressing HCC cells. Furthermore, knockdown of C1GALT1 resulted in the suppression of this activity. 

## 9. Interaction of Core-1 Synthase and Galectin in Prostate Cancer

Tsai et al. studied 22Rv1-derived sublines showing elevated expression of C1GALT1, ST3GAL1, and ST6GALNAC4, along with reduced expression of B3GNT6 (Core-3 synthase) and GCNT3 (Core-2 synthase) [51]. Because T-antigen-expressing glycoproteins act as receptors for galectin-4, they were the candidate glycoproteins studied in the progression of prostate cancer (PCa). The overexpression of galectin-4 in PCa cell lines, along with the altered O-glycosylation, promoted cancer metastasis to lungs. This phenotypic change was observed along with the induction of an EMT-like phenotype. Furthermore, these PCa cell lines lacked ligands for EGFR, HER3, and IGF1R, and thus galectin-4 mediated receptor tyrosine kinase (RTK) phosphorylation was found to be the key interaction for promoting cancer metastasis. This interaction was facilitated by the O-linked glycans on RTK. In a recent study by Tzeng et al., the relevance of O-glycosylation on castration-resistant prostate cancer (CRPC) was explored [52]. This study reported the role of sialylated T-antigen on metastasis to lymph node via galectin-4 interaction, which resulted in MYC activation. In addition to galectin-4 modification by sialyl-Tn in metastasis, it is also an essential interaction in CRPC development. Thus, O-glycosylation changes triggered malignant transformations that resulted in metastatic behavior and castrate resistance of cancer cells. These findings not only suggest the relevance of C1GALT1 in this particular cancer, but also highlights the role of the galectin family protein in the development of disease. 

## 10. Core-1 Synthase Behaves Differently in Different Cancers

Chou et al. explored the role of C1GALT1 in ovarian cancer [53]. The elevated expression of C1GALT1 was associated with poor survival in ovarian cancer patients. C1GALT1 knockdown resulted in decreased cell growth, migration, and sphere formation in a variety of ovarian cancer cell lines. Since C1GALT1 participates in an important step of O-glycan synthesis, this makes the enzyme very important for a multitude of cancerous processes. Lin et al. investigated the role of C1GALT1 in head and neck squamous carcinoma (HNSCC). C1GALT1 was found to be an independent attributor for poor OAS of HNSCC patients [54]. C1GALT1 affected cell viability, proliferation, invasion, tumor growth, and lung metastasis in HNSCC cells. The authors identified the mechanism by which C1GALT1 regulates tumorigenesis in HNSCC. EGFR O-glycans were found to be affected by C1GALT1 knockdown and this further regulated EGF-EGFR binding affinity to promote tumorigenic behavior. The authors conducted computational docking studies to identify an inhibitor, itraconazole, which degrades C1GALT1 through a proteosomal mechanism. This inhibitor mimicked the phenotypes obtained for C1GALT1 loss. In a mouse model, C1GALT1 loss reduced tumor burden in HNSCC. 

Zhang et al. studied the role of C1GALT1 in radioresistance of esophageal cancer cells [55]. They discovered that the loss of C1GALT1 resulted in increased radiosensitivity of esophageal cancer cells. In addition, oncogenic features, such as invasion, were increased in the radiation-sensitized cells. This was correlated with C1GALT1 expression, which increased upon radiosensitization. The loss of C1GALT1 resulted in reduced migration of cells both in the presence and in the absence of irradiation. As O-glycan on β1-integrin is influenced by C1GALT1, this study also identified β1-integrin as one of the important oncoglycoproteins regulated by C1GALT1 knockdown. Blocking β1-integrin and downstream signaling events such as FAK resulted in apoptosis in esophageal cancer cells. This suggests that radiosensitivity of these cancer cells is increased upon truncating O-glycans. Altogether, the participatory role of O-glycosylation on the β1-integrin driven signaling pathway was exhibited on cellular radiosensitization. 

## 11. Unique Study on Core-3 Synthase in Pancreatic Cancer

A recent report from Radhakrishnan et al. demonstrated the suppressive role and function that Core-3-mediated glycosylation has on pancreatic cancer growth and metastasis to lymph node and peritoneum [56]. Clinical patient samples were used to perform gene expression analysis from normal pancreata, matched liver metastatic sites, and tumor tissues to reveal the differential expression pattern of Core-3 synthase (B3GnT-6), as well as additional glycosyltransferases. Herein, they reported that decreased expression of Core-3 synthase is found in pancreatic cancer tumor tissues, as well as pancreatic cancer cell lines (Capan-2 and FG). Using re-expression analysis of Core-3 in these cell lines, the authors were able to determine that Core-3 synthase-mediated glycosylation is essential in reducing cell proliferation and actin polymerization, and decreases the ability of cells to invade and migrate as measured by invasion and migration assays. The authors concluded that Core-3 mediated glycosylation of MUC1 (a well-characterized oncoprotein), FAK, and integrins largely constitute the behavior in which PC growth and metastasis occurs. Core-3 synthase in PC is largely unexplored; however, it warrants further investigation because of the significance Core-3 has on delaying growth and metastasis.

## 12. Implications of Core-3 Synthase in Colon and Colorectal Cancer

**Figure 3:**
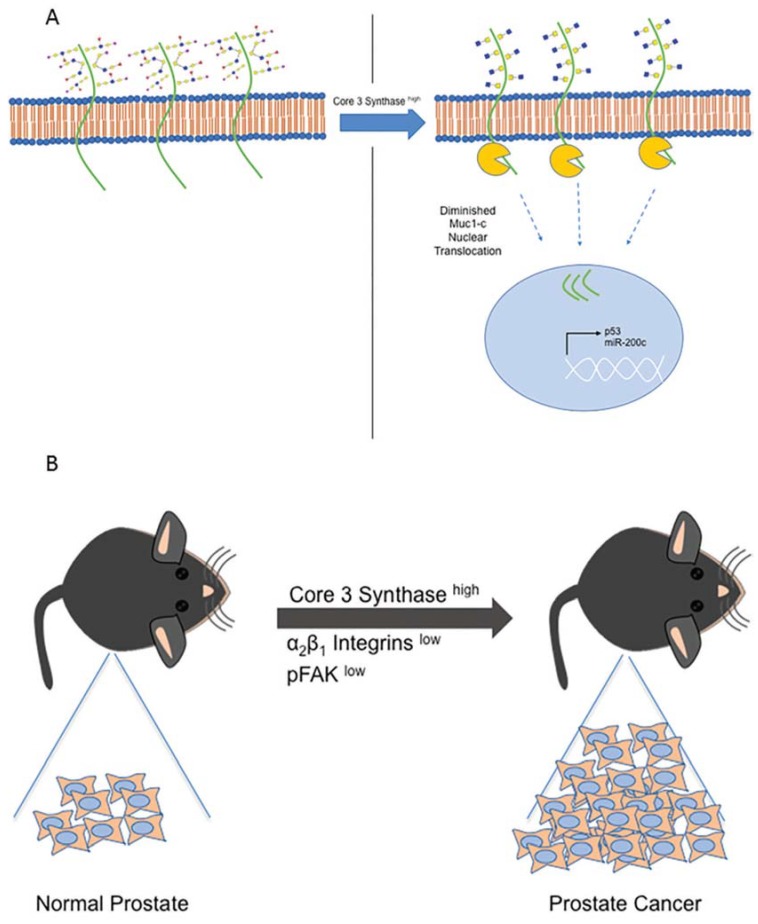
The schema here show the role of Core-3 synthase in the regulation of biological processes in colon cancer (**A**) and prostate cancer (**B**). Core-3 synthase (B3GNT6) negatively regulated the role of MUC-1 in colon cancer. An increase in B3GNT6 reduced MUC-1 C-terminus translocation to nucleus, and increased p53 and miR-200c expression. This phenomenon was observed in an in vitro model of colon cancer. Meanwhile, in prostate cancer, an in vivo study exhibited role of increased Core-3 synthase. To this end, Core-3 synthase overexpressing cells were orthotopically implanted into the prostate of mice and were shown to reduce the tumor burden, along with the expression of α2β1 integrin and pFAK.

Core-3 structures have been primarily associated with the tissues of the stomach and colon. Iwai et al. cloned and characterized the B3GNT6 gene responsible for Core-3 synthase [57]. The investigators worked on its role in colon cancer progression. To identify the role of Core-3 synthase, this group synthesized an antibody that specifically recognized B3GNT6 expression. The study identified that the expression of B3GNT6 (Core-3 synthase) decreased in gastric and colon cancerous tissues as compared to the normal tissues. In familial adenomatous polyposis (FAP), which represents colon cancer predisposition syndrome, B3GNT6 expression decreased from stage C1 to C5, with no staining at all in C4 and C5 stages. Furthermore, Caco-2 cells were found to have higher expression of B3GNT6 upon differentiation, suggesting that the gene is lost as the cells turn cancerous. The B3GNT6 gene was stably expressed in CRC cell lines and was found to reduce migration in an in vitro setting. The authors conducted in vivo studies by injecting B3GNT6-overexpressing cells through mice tail veins. They observed overall decreases in lung metastasis. Although this study highlights the role of the B3GNT6 gene on colon cancer, the underlying mechanism or specific glycoprotein involved in this process is not yet identified. However, this is the first instance to demonstrate the role of Core-3 synthase in colon cancer. Another study delineated the role of B3GNT6 in colon cancer [58]. A loss of B3GNT6 was associated with lymph node metastasis, distant metastasis, and poor OAS. In addition, it was observed that Core-3 regulated the plasticity of the EMT and mesenchymal-epithelial-transition in CRC. The overexpression of B3GNT6 in colon cancer cell lines resulted in diminished MUC1-C nucleus translocation, elevated p53 gene transcription, and the activation of miR-200c (Figure 3A). The relevance of B3GNT6 has also been elucidated in a Core-3 synthase KO animal model [59]. Core-3 synthase KO animals, when treated with Dextran Sodium Sulfate (DSS), induced colorectal tumorigenesis. The KO animal model had phenotypes of increased epithelial cell proliferation even in the absence of azoxymethane (AOM) and DSS. As the downregulation of B3GNT6 has been observed in human colon carcinoma, this study dictates a possible predisposition of the epithelium towards tumorigenesis. Additionally, the altered expression of β-catenin/TCF-4, c-myc, and cyclin D1 was observed during chemical-induced carcinogenesis, suggesting a possible role of B3GNT6 KO in human colorectal carcinoma. 

With the advent of mass spectrometry, glycosylation studies have increasingly become more thorough and impactful [60]. The impact of O-glycosylation in colon cancer used mass spectrometry to identify glycan structural differences on MUC2 in carcinoma, transitional mucosa, and adjacent normal tissue. The group found increased expression of STn on cancerous samples. Surprisingly, there was a higher abundance of sialylated Core-3 structures in colon cancer as compared to the normal adjacent tissue. Although this exhibits discrepancy with respect to the previous studies reported on Core-3, it can be explained because tissues from three patients were used instead of tissues from a larger cohort. In addition, the authors associated the presence of sialylated Core-3 structures mainly to MUC2, independent of the tissue or malignancy. 

## 13. Core-3 Synthase Overexpression Study in Prostate Cancer

While many knockdown studies for Core-3 synthase have been reported for other cancers, the overexpression analysis of B3GNT6 was conducted in PCa [61]. In this study, PC3 and LNCaP cell lines were utilized for overexpressing B3GNT6, resulting in reduced migration and invasion in an in vitro assay, suggesting reduced tumorigenesis. Additionally, Core-3 synthase overexpressing cells, when orthotopically implanted into the prostate of nude mice, resulted in reduced tumor burden and almost negligible metastasis to lymph nodes. B3GNT6 overexpressing cells had reduced amounts of α2β1 integrins, which further reduced cell migratory potential. These cells also had reduced FAK phosphorylation, suggesting an impairment of the signaling pathway. While α2β1 integrins were found to be a critical component for reducing cell migration and tumorigenesis, the exact mechanism by which α2β1 integrins are reduced in Core-3 synthase expressing cells remains unknown (Figure 3B). Thus, overall, there are fewer studies pertaining to Core-3 synthase on PCa and further in-depth analysis is required to decipher its role and mechanism in this cancer type. 

## 14. In Vitro and In Vivo Role of ST6GalNAc-I in Ovarian Cancer

In many instances, truncated glycosylation has been associated with ovarian cancer. A recent study by Starbuck et al., demonstrate the unique association between ovarian cancer and the population of cancer stem cells containing TACA [62,63]. The cell surface antigen CD133+ cell is composed of the stem cell niche and is considered as a regulator of cancer progression. These cells were compared with sialyl-Tn+ ovarian cells. Sialyl-Tn+ ovarian cancer cells had increased colony formation and tumor-sphere formation compared to CD133+ cells. Treatment of these cells with the anti-sialyl-Tn antibody drug conjugate (ADC) S3F-CL-MMAE resulted in reduced stem cell forming ability. In addition to the in vitro assays, an in vivo xenograft model resulted in reduced tumor burden in the presence of ADC S3F-CL-MMAE [63]. 

## 15. Diagnostic Influence of ST6GalNAc-I in Pancreatic Cancer

Truncated O-linked glycosylation has been considered as a hallmark of pancreatic cancer. In a study conducted on various pancreatic tissues, including normal, primary tumor, and liver metastatic tissues, sialyl-Tn levels were found to be upregulated in the primary tumor and liver metastatic tissues [64]. The authors also studied different stages of PC and found that sialyl-Tn was not observed in the PanINs, suggesting its expression occurs in later stages of pancreatic cancer. Because mucins have been known to be associated with aggressive PC, the antigen expression profile was assssed. Mucin analysis of these samples exhibited sialyl-Tn expression on MUC1 in primary tumors but not normal pancreas tissue. Sialyl-Tn+ MUC1 can thus be used to increase specificity to diagnose PC.

## 16. ST6GalNAc-I Dependent Mucin Glycosylation in Gastric Cancer

Sialyl-Tn is one of the major glycan structures expressed on the surface of tumor cells. It is mainly synthesized by ST6GalNAc-I and secondly by the ST6GalNAc-II enzyme. Studies on ST6GalNAc-I have been focused primarily on calculating the enzyme activity in various cell types and tissues. Marcos et al. studied the expression of this enzyme in four gastric carcinoma cell lines: MKN45, KATOIII, GP202 and GP220 [65]. Herein, sialyl-Tn expression was found to be lower than that observed in the control cell line, K562. This was further correlated with the lower amounts of sialyl-Tn antigen found in gastric carcinoma cell lines. Another group studied the gastric cancer cell line MKN45, which expresses very high levels of ST6GalNAc-I and, consequently, higher sialyl-Tn levels. In this cell line, loss of ST6GalNAc-I resulted in reduced proliferation, migration, invasion, and reduced STAT5b phosphorylation and IGF-1 expression [66,67]. Ozaki et al. reported increased peritoneal metastasis of sialyl-Tn-expressing gastric carcinoma cell lines when transplanted into nude mice [68]. Marcos et al. later studied the substrate specificity of ST6GalNAc-I and ST6GalNAc-II by using synthetic glycoconjugates and peptides that mimic some of the commonly expressed mucins. MUC2 has been shown to be the major carrier of sialyl-Tn on intestinal metaplasia and gastric cancer [69]. Moreover, ST6GalNAc-II was found to decorate sialic acid onto MUC-2 and MUC-4 mucinous peptides as efficiently as ST6GalNAc-I, suggesting a secondary role of this enzyme in synthesizing sialyl-Tn epitope. These enzymes were expressed in gastric carcinoma cells and synthesized sialyl-Tn epitopes with ST6GalNAc-I majorly forming this epitope as compared to the ST6GalNAc-II [70,71,72]. 

TACA associated with gastric carcinoma suggests elevated expression of Tn, sialyl-Tn, and T-antigen. In a cohort of patients studied for gastric carcinoma, Tn (96.1%) and sialyl-Tn (89.6%) were found to be expressed. This study highlighted the role of MUC1 VNTR polymorphism with the expression of truncated carbohydrates sialyl-Tn, Tn, and T-antigen [73]. The length-dependent polymorphism of MUC-1 minimally regulated sialyl-Tn and Tn antigen expression whereas T-antigen expression was highly influenced. High levels of sialyl-Tn were also observed in the MST6-I cell line, suggesting its role in a malignant phenotype [74]. These cells had significantly diminished homotypic cellular contacts. Oncogenic transformations were also noted due to the increased expression of lamellipodia and filopodia in MST6-I cells as compared to the mock-transfected cells. MST6-I cells had large protrusions as compared to mock-transfected cells and exhibited increased adhesion, migration, and invasion. This phenotype agrees with the observation mentioned above, wherein a loss of ST6GalNAc-I resulted in reduced adhesion, migration, and invasion. Since mucin glycosylation plays a critical role in cancer progression, analysis of various mucins has been carried out in different cancer types using the in situ proximity ligation assay [75]. High sialyl-Tn levels have been found on MUC1 and MUC2, and lower on MUC6 and MUC5Ac. The proximity ligation assay determined a direct association between different glycoforms and mucins in cancer samples. 

## 17. ST6GalNAc-I Based Immune Therapy in Breast Cancer

Several studies have reported varying levels of sialyl-Tn found in breast cancer patients. Schmitt et al. found a lower frequency of sialyl-Tn-positive cells in breast invasive ductal carcinoma as compared to ductal carcinoma [68]. Additionally, sialyl-Tn expression was correlated with poor survival rate, loss of estrogen receptor, and c-erb-B2 oncogene activation [76,77,78,79,80,81]. Because earlier studies lacked direct evidence of the role of sialyl-Tn in breast cancer, Julien et al. overexpressed ST6GalNAc-I into multiple breast cancer cell lines [82,83]. As endogenous levels of ST6GalNAc-I in these cell lines are either basal or absent, the direct role of sialyl-Tn was studied through the overexpression of ST6GalNAc-I. In one of the cell lines, sialyl-Tn-positive cells possessed decreased growth and higher mobility. The sialyl-Tn+ cell line, when injected into SCID mice, resulted in increased tumor growth, thus suggesting potential implications of sialyl-Tn on breast cancer progression. The same group later used a T47D breast carcinoma cell line and forced expression of ST6GalNAc-I [84]. Its expression resulted in Sialyl-Tn synthesis on the cell surface. The enzyme activity of ST6GalNAc-I was higher in transfected cells as compared to the mock. These cells were observed to have decreased cell growth, lower cell adhesion on ECM components, and higher mobility. This is in agreement with the results found on other breast cancer cell lines. The sialyl-Tn epitope was mostly found on higher molecular weight proteins, including MUC1, and thus it can be speculated that anti-adhesive properties of these cells are due to aberrantly glycosylated MUC1.

Sewell et al. studied sialyl-Tn expression on breast cancer cells and correlated its expression with the transcript levels of ST6GalNAc-I [85]. ST6GalNAc-II levels did not correlate with the sialyl-Tn expression in many of the patient samples. However, ST6GalNAc-I was found to be distributed in the Golgi stack, which explains its competitive edge over Core-1/Core-2 synthase resulting in the synthesis of sialyl-Tn. This study also resulted in the generation of CHO cells expressing sialyl-Tn decorated MUC-1. It is speculated that glycol-engineered MUC-1 might be useful in developing immunotherapy against sialyl-Tn-containing glycoproteins expressed in tumors. Sialyl-Tn-decorated MUC-1 based immunotherapy has gained attention recently [86]. Multiple immunogens based on sialyl-Tn-modified KLH (Theratope), sialyl-Tn-modified MUC-1 peptide, and MUC-1 protein have been used to eradicate tumor progression from a MUC-1 transgenic mouse model. Amongst these three immunogens, theratope has been shown to significantly delay tumor progression. The underlying glycoprotein resulting in delayed tumor progression was identified as osteopontin. A potential candidate for therapeutic interventions in the treatment of metastatic breast cancer is sialyl-Tn-KLH [87]. Sialyl-Tn-KLH, along with endocrine therapy, resulted in increased overall time to progression (TTP) and OAS of patient groups in comparison to the unconjugated KLH. As sialyl-Tn is the most studied glycotype in cancer, recent advancements in the field have resulted in the generation of sialyl-Tn-based immunotherapy. This is a huge success in the field of cancer glycobiology and encourages various research groups to identify novel glycan targets for improving cancer therapy.

## 18. Mechanistic Insights of ST6GalNAc-I in Different Bladder Tumor

Sialyl-Tn- and sialyl-Tn/S6T-expressing bladder tumors were treated with bacillus Calmette-Guerin (BCG) [88,89]. Lower recurrence rates of sialyl-Tn-positive tumor cells were observed post-BCG treatment. Moreover, sialyl-Tn expressing cells had pronounced BCG-dependent adhesion, internalization, and apoptosis. To test glycosylation variations in bladder tumors, a novel approach of utilizing cancer cell lines with different genetic backgrounds was tested [90]. Herein, T24, 5367, and HT1376 cells were subjected to hypoxic conditions and they were found to express elevated levels of sialyl-Tn as compared to the normal control. Glycoproteomics identified MUC16 as the common glycoprotein expressing sialyl-Tn in all the three cell lines. Although the role of MUC16 in bladder tumors is unknown, this study is the first to report MUC16 as the sialyl-Tn carrier protein [91]. 

Costa et al. measured sialyl-Tn expression in muscle-invasive bladder cancer (MIBC), a highly lethal subtype of bladder cancer [92]. The authors found elevated levels of sialyl-Tn in MIBC when compared to non-MIBC. Sialyl-Tn expression was also associated with poor survival of MIBC. This study illustrated a novel approach to target sialyl-Tn-positive bladder tumor cells by targeting the PI3K/Akt/mTOR pathway in a mouse model with a BBN-induced tumor. In the presence of an mTOR inhibitor (rapamycin), sialyl-Tn expressing cells reduced, respectively. In circulating tumor cells and metastatic lesions of bladder cancer, a higher expression of sialyl-Tn has been reported. This is indicative of a novel approach of isolating and characterizing bladder tumor cells rich in the sialyl-Tn glycan structure [93]. 

## 19. Clinical Implications of ST6GalNAc-I in Colon Carcinoma

As seen in other cancers, sialyl-Tn expression has been believed to be higher in colon carcinoma and is correlated with its recurrence [94,95]. The differential expression of sialyl-Tn has been seen in adenomas and carcinomas with varying localization in a Chinese population [96]. The immunohistochemistry-based prognostic role of sialyl-Tn has been studied in 116 patients. Sialyl-Tn expression was mild in healthy tissues and increased significantly in tumor tissues and transitional colorectal tissues [97]. 

While sialyl-Tn levels have been studied in various tissues, its correlation with ST6GalNAc-I has not been reported in colon carcinoma. Enzymatic activity determines glycan expression on the cell surface, thus ST6GalNAc-I activity was measured in various tissues [98]. However, no correlation between the enzyme activity and sialyl-Tn expression was found, suggesting transcriptional regulation of sialyl-Tn. Because the expression of glycans on cell surfaces occurs via Golgi-specific intracellular process of post-translational modification, in vitro studies did not mimic the in vivo conditions. This proposition may explain the discrepancy in ST6GalNAc-I activity and sialyl-Tn expression in colon adenocarcinoma [99]. 

As sialyl-Tn has been a prominent marker in the study of colon and other cancers, various antibodies have been generated to quantify its expression. An antibody, 3P9, was generated by An et al. to look at the expression of sialyl-Tn in LSCSTn+ and LSBSTn- cells [100]. In a xenograft mouse model, administration of this antibody resulted in decreased sialyl-Tn+ tumor burden. Additionally, 3P9 induced apoptosis in sialyl-Tn+ cells and inhibited the migration and proliferation rate of sialyl-Tn-expressing cells. 

In addition to colorectal carcinoma, ST6GalNAc-I expression has been found to be elevated in cancer stem cells (CSC) populations [101]. The authors indicated an association of CSC markers with higher incidence of sialyl-Tn expression in these CSCs. Cells overexpressing ST6GalNAc-I resulted in higher sialylation on CD44 and activated the Akt pathway. The activation of the Akt pathway was subsidized by blocking galectin-3, which recognizes sialyl-Tn on cell surfaces [102]. 

## 20. MUC1 Regulation by ST6GalNAc-I in Ovarian Carcinoma

Aberrantly glycosylated forms of mucins participate in a variety of cancer diseases. One such mucin, MUC1, is known to play a key role in ovarian cancer [103]. MUC1 is highly expressed in ovarian carcinoma but not in ovarian surface epithelium (OSE) and serous cystadenomas. 5E5 antibody, which recognizes Tn/sialyl-Tn epitopes of MUC1, was highly expressed in ovarian carcinoma, suggesting a role of truncated glycans. An association of sialyl-Tn with MUC16 has been noted in differentiating endometriosis from ovarian carcinoma with 44% sensitivity and 100% specificity. MUC1 and MUC4 levels have also been reported in mucinous ovarian carcinoma, but the implications of glycosylation on these mucins has not been explored in ovarian carcinoma. The epithelial layer of the ovarian endometriosis has been shown to also express Tn/sialyl-Tn epitopes, further suggesting possible implications in tumor development [104]. Tissue and serum specific sialyl-Tn levels have been inversely related with patient survival, as reported cumulatively by various authors [68,105]. 

## 21. In Vitro and in Vivo Role of ST6GalNAc-I in Bladder Tumor

Sialyl-Tn’s role has been explored extensively in many carcinomas; however, the role of ST6GalNAc-I in bladder cancer is not well understood. Higher expression of sialyl-Tn was observed in advanced stages of bladder tumor but not in the control group [106]. Sialyl-Tn expression was also associated with the mucin glycoproteins MUC1, MUC2, and mucin antigen of the urinary bladder (MAUB) derived from bladder tumors. A partial direct correlation between the Ki-67 staining and sialyl-Tn expression has also been observed. Higher levels of sialyl-Tn have been observed in the location of invasion corresponding to the basal and muscle layers, suggesting a higher prevalence in the malignancy of bladder tumors. Various bladder cancer cell lines, such as HT1376, 5637, T24, and MCR, were associated with lower incidences of the sialyl-Tn antigen. Overexpression of ST6GalNAc-I in the MCR cell line increased sialyl-Tn expression. However, a minimal proliferation of cancer cells was observed with an increase in invasion and migration. To study the in vivo role of sialyl-Tn antigen, Bernardo et al. developed a xenograft model of sialyl-Tn expressing bladder tumor with a higher incidence rate [107]. Sialyl-Tn expression correlated with the higher proliferation index based on p53 and Ki-67 biomarkers. Successful conversion rate of the xenograft model was exhibited, and sialyl-Tn expression was retained in the primary bladder tumor of second- and third-generation xenografts. This study illustrated the development of an animal model towards the study of sialyl-Tn-positive bladder tumor. 

Another group studied the role of immune cells on the function of sialyl-Tn-expressing bladder tumor cells [108]. These cells were correlated with CD1a expression, a marker for immature DCs. Additionally, sialyl-Tn levels were also in coherence with pro-inflammatory markers, such as IL-12 and TNF-alpha. Sialyl-Tn-expressing MCR bladder tumor cells adhered to immature DCs, which resulted in inhibition of DC maturation. LPS treatment failed to stimulate DC cells, suggesting resistance to maturation post co-culture with sialyl-Tn+ cells. A majority of sialyl-Tn was associated with CD44 on these MCR-sialyl-Tn+ cells. The blockade of CD44 and MUC1 on the MCR-sialyl-Tn+ cells resulted in the elevated expression of MHC-II, IL-12, and TNF-alpha, suggesting the maturation of DCs. In addition, monocyte-derived DCs, which had phagocytosed MCR-sialyl-Tn+ cells, resulted in lower activation of T cells as measured by CD69 expression. Such DCs promoted lower levels of Th1 cells and directed T cells towards a FoxP3high and IFNgamma low profile. 

## 22. Identifying Splice Variant of ST6GalNAc-I in Prostate Cancer

PCa has an overall low survival rate when the tumor becomes castrate-resistant (CR). CRPCa involves a role of the androgen receptor (AR), including its signaling and transcriptional regulation. A recent study highlighted the increases in ST6GalNAc-I levels in the AR-induced PCa cells [109]. This study further showed increases in STn levels on AR positive PCa cells. Moreover, a novel finding exhibited the presence of a new splice variant of ST6GalNAc-I (55kDa) in these AR-induced cells as compared to the steroid deprived. This splice variant possessed enzyme activity as its over-expression in PCa cells increased STn levels. The shorter form, in addition to the longer form, of ST6GalNAc-I induced a mesenchymal like phenotype, less cell adhesion, and more migration using in vitro assays. Overexpression of STn in PCa cells showed reduced tumor growth in subcutaneous implantation in nude mice [110].

## 23. Tumor Suppressor Role of C2GnT/GCNT in Pancreatic Cancer

In this study, C2GnT transfected the expression of sLex using various antibodies, like CSLEX-1, CC49, SM-3 and HMFG-2, were compared in pancreatic cancer cell lines [111]. sLex expression was found to be higher in cell lines transfected with C2GnT and alpha-1,3 fucosyltransferase. Because C2GnT has been known to co-localize with ST6GalNAc-I and -II, these data indicated low or no expression of Neu5Acα2,6GalNAc. Furthermore, C2GnT has also been known to co-localize with ST3Gal-I, which results in the abrogation of the sialylated core-1 structure on MUC1. An isoform of C2GnT, GCNT3, has been shown to overexpress in pancreatic cancer and was associated with poor patient survival [112]. Higher expression of GCNT3 was noted in p48^Cre/+^-LSL-Kras^G12D/+^ mice as compared to the normal pancreas. GCNT3 was also associated with increased mucin biosynthesis. This study reported on the first ever inhibitor for GCNT3, talniflumate, which competes with the GCNT3 recognition site for Galβ1,3GalNAc. Talniflumate reduced the protein levels of GCNT3 and mucin and suppressed the mRNA levels of GCNT3. Talniflumate, along with the EGFR inhibitor gefitinib, reduced GCNT3 and mucin expression. This drug combination also resulted in the reduction in cellular proliferation and spheroid formation. GCNT3 upregulation was also seen when pancreatic cancer cell lines were treated with rosemary extracts [113]. This upregulation was correlated with the antitumor activity. In silico analysis exhibited the role of miR-15b in the regulation of GCNT3, which has been shown to also be involved in anti-tumor activity of rosemary extracts. 

## 24. Implications of GCNTs in Colorectal Cancer

sLex and sLea are associated with malignancy [114]. Thus, it is important to identify the expression levels of C2GnT enzymes involved in the synthesis of precursor glycan to both sLex and sLea. Shimodaira et al. studied the levels of C2GnT1 or GCNT1 in colorectal carcinoma and found its level to be higher in 63% of the patient samples [115]. Patients expressing C2GnT1 had more lymphatic and venous invasion as compared to those patients having low levels of C2GnT1. On the contrary, C2GnT-3 levels were reported as low in colorectal carcinoma when compared to the normal colon tissues. Because Core-1 is expressed constitutively in malignancy, this study identified the relevance of other enzymes that act as successors to Core-1 synthesis. In another study, C2GnT-M levels were independently correlated with C2GnT-L levels, which predominantly catalyzed Core-2 structures and not Core-4 and I antigen [116]. C2GnT-M reduced invasion in matrigel and cell adhesion of collagen-IV and fibronectin. Paxillin specific tyrosine phosphorylation at Y118 was significantly reduced in C2GnT-M overexpressing cells. C2GnT-M transfected HCT116 cells, upon orthotopic implantation in nude mice, resulted in reduced tumor burden, suggesting an in vivo role of C2GnT-M on tumorigenesis. Lower levels of GCNT3 have significantly been shown to result in higher rates of relapse in patients with colorectal carcinoma [117]. Moreover, the effect of chemotherapeutic agents, such as 5-FU, bortezomib, and paclitaxel, results in variable expression of GCNT3. This further indicates a role of this enzyme in carcinogenesis inhibition and a probable role in the development of anticancer therapy. 

Other than GCNT1 and GCNT3, GCNT2 primarily synthesizes I antigen and is not involved in O-glycosylation. However, because multiple studies suggests its important role in CRC, we chose to describe those studies here [118]. GCNT2 expression is regulated by EGF, bFGF-t, and miR-199 in the regulation of colon carcinoma. This enzyme results in increased survival, proliferation, and invasion of tumor cells. This has been shown to promote tumorigenicity by upregulation of AKT and ERK signaling. miR-199 inversely regulates C2GnT-2 and inhibits cancer cell motility and invasion. GCNT2 expression is mostly downregulated in CRC and is epigenetically regulated [119]. This regulation is dependent on the DNA methylation status of its TSS. Hypomethylation in the GCNT2 variant 2 has been correlated with lymph node metastasis in CRC. 

## 25. Mechanistic Insights of C2GnT1/GCNT1 in Prostate Cancer

As the ligands for P-, L-, and E-selectins all participate in cancer metastasis, the identification of the underlying gene involved in this process is indispensable. To study this, the role of C2GnT gene was explored in PCa [120]. In particular, a specific polymorphism in C2GnT (valine at 152) was predisposed to PCa. C2GnT was found to be overexpressed in PCa compared to the normal prostate. However, the in vitro catalytic activity between the valine and isoleucine form resulted in compared catalytic activity. This in vitro catalytic activity did not directly reflect the in vivo functional significance. 

TNFα, a proinflammatory cytokine, has been shown to upregulate C2GnT1 along with other genes [121]. In PCa, TNFα induced motility and invasiveness owing to the upregulation in many glycosyltransferase genes, including C2GnT1. C2GnT1 expressing PCa cells have polylactosamine terminated MUC1 chains, which results in the attenuation of NK-PCa cells interactions. This attenuation increases the survival rate of PCa cells in host circulation. Furthermore, O-glycan-terminated MUC1 reduces the adhesive characteristics of PCa cells [122]. When C2GnT-1-overexpressed PCa cells were injected into nude mice, it resulted in larger tumors compared to the vector control [123]. C2GnT1-expressing PCa cells possessed higher binding to prostate stromal cells and released significantly higher amounts of growth factors, such as HGF, FGF, and VEGFA. The levels of these growth factors were regulated by galectin-3 expression, which was increased in C2GnT1 expressing PCa cells. C2GnT1 over-expression also results in altered O-glycosylation of PSA (prostate specific antigen), PAP (prostatic acidic phosphatase), and MUC1 in PCa cells [124]. 

## 26. Immune Evasion by C2GnT in Bladder Tumor

C2GnT synthesizes the Core-2 structure on glycoproteins. In this study, MUC1 was found to express Core-2 extended polylactosamine glycans, primarily in C2GnT-expressing bladder tumors [125]. Modified MUC1, due to the overexpression of C2GnT, results in immune evasion of tumor cells by NK cells that further results in a longer half-life of tumor cells in circulation. Moreover, C2GnT expressing bladder tumors contain polylactosamine chains that are involved in the NKG2D-MICA interaction that is regulated by galectin-3 [126]. This interaction further stimulates NK cell activation. Thus, C2GnT decorated extended O-glycans participate in immune evasion of bladder tumor cells. As there are not many studies focusing on this aspect, the underlying molecular mechanisms resulting in these biological events are unknown and are subjected to further analysis. 

## 27. Conclusions

In the present review, we have compiled results from the last three decades covering all the major highlights on the glycosyltransferases associated with various cancers. In cancer, the role of O-glycosylation has long been known, but a comprehensive analysis covering the key enzymes involved in its extension and truncation is lacking; thus, this review pays tribute to the critical enzymatic steps associated with cancer progression. Here, the effects of glycosylation on various markers have been shown to influence proliferation, migration, invasion, and metastasis. Not only limited to in vitro studies, various in vivo animal models have been explored to identify their phenotypic effect on tumorigenesis. 

Various studies have been illustrated that covered the direct effect of glycosyltransferases on tumor progression. Recent studies have exhibited mechanisms pertaining to glycosylation-induced cancer. In this regard, the structure-function analysis due to the loss of specific glycosyltransferase has not been given much emphasis. Thus, applying the knowledge of glycomics to the field of cancer, glycobiology will act as a stepping-stone for understanding the precise role of glycan structures on tumorous conditions. This is an important area of study; further research will reveal the fundamental changes that a loss of glycosyltransferase has on inducing cancerous condition. 

Additionally, understanding the differential regulation of glycosyltransferases will result in the identification of biomarkers for early cancer detection. In this direction, various glycoproteins such as prostate specific antigen (PSA), MUC16, aberrantly glycosylated MUC1 and CA19-9, including others, have been used historically to detect various cancers. Thus, targeting glycans in addition to the protein backbone will pave way to a deeper knowledge towards biomarker discovery. Furthermore, understanding novel aspects of glycan synthesis in normal versus tumorous condition will permit design of targeted therapies including immunotherapies. Since the glycan signature of a person will depend on the overall cellular health, studying this aspect will thus assist in developing tools for personalized medicine.
